# Classification of head and neck cancer from PET images using convolutional neural networks

**DOI:** 10.1038/s41598-023-37603-1

**Published:** 2023-06-29

**Authors:** Henri Hellström, Joonas Liedes, Oona Rainio, Simona Malaspina, Jukka Kemppainen, Riku Klén

**Affiliations:** 1 Turku PET Centre, University of Turku and Turku University Hospital, Turku, Finland; 2 Department of Clinical Physiology and Nuclear Medicine, Turku University Hospital, Turku, Finland

**Keywords:** Molecular medicine, Oncology, Mathematics and computing

## Abstract

The aim of this study was to develop a convolutional neural network (CNN) for classifying positron emission tomography (PET) images of patients with and without head and neck squamous cell carcinoma (HNSCC) and other types of head and neck cancer. A PET/magnetic resonance imaging scan with ^18^F-fluorodeoxyglucose (^18^F-FDG) was performed for 200 head and neck cancer patients, 182 of which were diagnosed with HNSCC, and the location of cancer tumors was marked to the images with a binary mask by a medical doctor. The models were trained and tested with five-fold cross-validation with the primary data set of 1990 2D images obtained by dividing the original 3D images of 178 HNSCC patients into transaxial slices and with an additional test set with 238 images from the patients with head and neck cancer other than HNSCC. A shallow and a deep CNN were built by using the U-Net architecture for classifying the data into two groups based on whether an image contains cancer or not. The impact of data augmentation on the performance of the two CNNs was also considered. According to our results, the best model for this task in terms of area under receiver operator characteristic curve (AUC) is a deep augmented model with a median AUC of 85.1%. The four models had highest sensitivity for HNSCC tumors on the root of the tongue (median sensitivities of 83.3–97.7%), in fossa piriformis (80.2–93.3%), and in the oral cavity (70.4–81.7%). Despite the fact that the models were trained with only HNSCC data, they had also very good sensitivity for detecting follicular and papillary carcinoma of thyroid gland and mucoepidermoid carcinoma of the parotid gland (91.7–100%).

## Introduction

Head and neck cancers are a heterogenous group of cancers located in the lips, oral cavity, nasal cavity and sinuses, throat (pharynx), salivatory glands, voice box (larynx), vocal cords, and the epiglottis^1^. Over 90% of them begin in the squamous cells lining the mucosal surfaces and therefore most of head and neck cancer patients are diagnosed with squamous cell carcinomas (HNSCC)^2^. Other possible head and neck cancer types are adenocarcinomas, adenocystic carcinomas, chondrosarcomas, mucoepidermoid carcinomas, acinic cell carcinomas and follicular, papillary, medullary and anaplastic carcinomas of the thyroid gland. Together, different head and neck cancers constitute the sixth most common type of cancer in the world, with over 930,000 new causes and 420,000 deaths estimated in 2020^3^.

Usual treatment options for head and neck cancers include surgery, radiation therapy, chemotherapy, and immunotherapy. During the treatment, it is necessary to have accurate information about the exact location of the cancer because the cancerous cells can spread by metastasizing locally or to the lymph nodes of the neck. Monitoring the patients after their treatment is also important due to their high risk of developing a second primary cancer^1^.

One option for examining head and neck cancer patients is a nuclear medicine imaging method known as positron emission tomography (PET). This method enables the detection of molecular events in human body, for example glucose consumption with ^18^F-fluorodeoxyglucose (^18^F-FDG) where glucose molecule is labeled with positron emitting ^18^F-radionuclide. With this radiotracer enhanced glucose consumption commonly detected in cancer tissues and in infectious or inflammatory processes can be detected with higher sensitivity than with conventional radiological imaging methods^4^.

However, interpretation of the results of a PET scan is both relatively difficult and very laborious. It is required that a specialized physician compares the PET images with the computer tomography (CT) or magnetic resonance imaging (MRI) images taken during a PET scan. The repetitive nature of the work can make it tedious especially if there are numerous cancer patients to be monitored, and the time of the medical professionals is often very limited under increasing workload pressure. Furthermore, several factors, including the time used, properties of the image processing software, and the experience and professional capabilities of the physician, can affect final conclusions.

This problem can be solved by creating a suitable convolutional neural network (CNN) that is designed to recognize cancer tumors from the patients’ PET images and classify them accordingly. Namely, a CNN is such an artificial neural network that processes visual imagery data by taking the spatial relationships between pixels or voxels into account. In similar research^5–9^, CNNs have successfully been applied for analyzing PET images of cancer patients.

## Materials and methods

### Dataset

The original dataset was retrospectively collected from 200 patients, who had been diagnosed with head and neck cancer, treated at Turku University Hospital and referred for a ^18^F-FDG PET/MRI scan at Turku PET Centre in Turku, Finland, during the years 2014–2022 to monitor their current tumor or to detect a possible recurrent tumor. The mean age of the patients was 62.8 years with standard deviation of 11.6 years (interval: 26–86 years), and their male–female sex ratio was 2.1. Out of the 200 head and neck cancer patients, 100 patients had no locoregional recurrence of cancer and are therefore considered as negative compared to the 100 positive patients who had a tumor visible in their PET/MRI images. 89 positive patients and 93 negative patients had been diagnosed with HNSCC and the rest 18 patients with other types of head and neck cancer.

**Figure 1:**
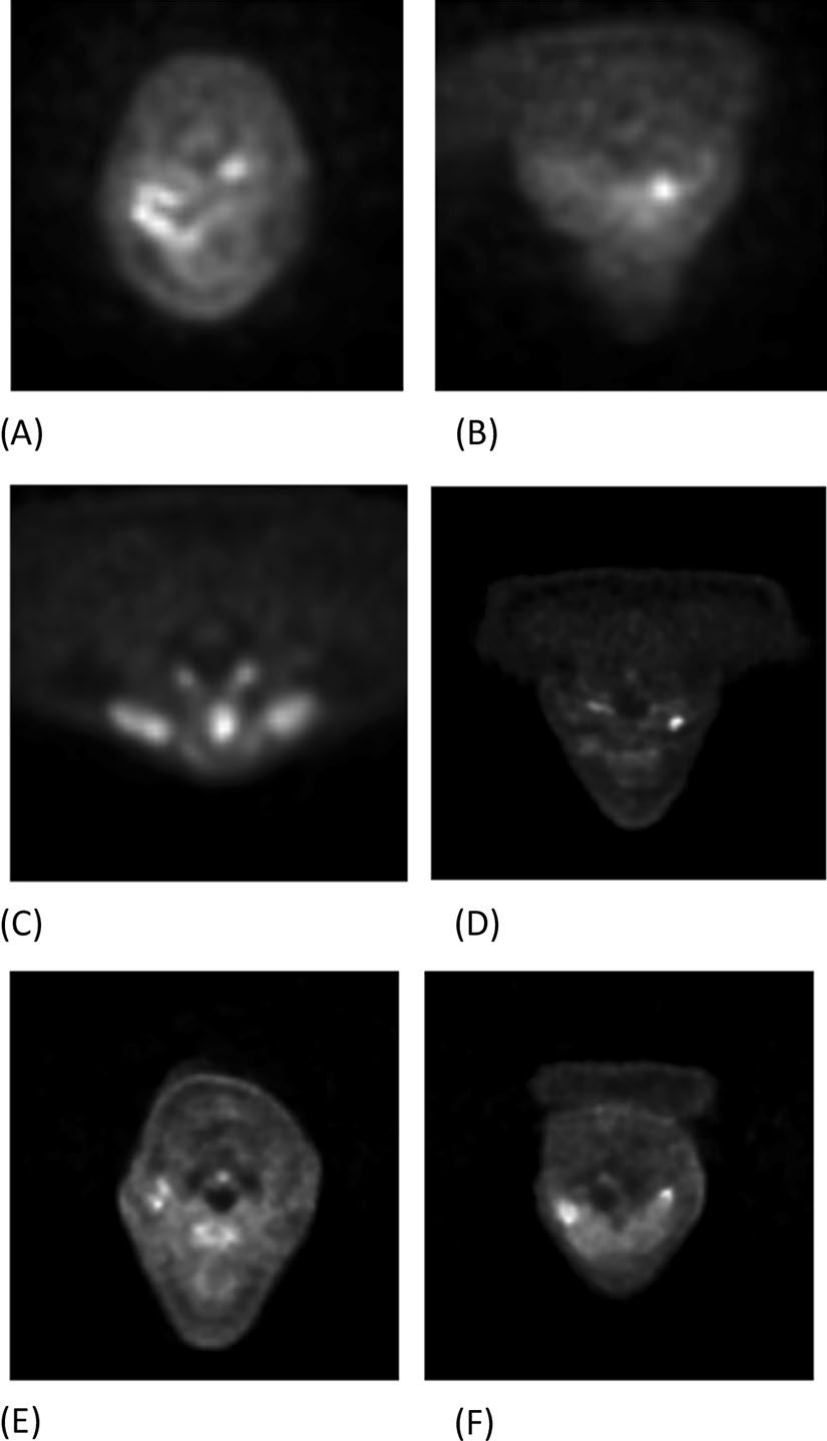
Examples of the positive transaxial PET slices before pre-processing, which show squamous cell tumor in the oral cavity (**A**), in the hypopharynx (**B**), in the larynx (**C**), in the left fossa piriformis (**D**), on the right tonsil (**E**), and on the left side of the gums in the lower jaw of the patient (**F**).

The ^18^F-FDG PET/MRI scans were performed with 3 T Philips Ingenuity TF PET/MR scanner (Philips Health Care) and SIGNA™ PET/MR with QuantWorks (GE Healthcare). Only the PET images were used in this study. The presence of the tumor was verified either with histological sampling or re-imaging scans. The possible re-imaging scans were excluded from the study so that exactly one PET image was considered from each patient. All the PET scans had been trimmed into the area from the forehead to the shoulders of patients to show only the possible locations of head and neck cancer tumors. See Fig. 1 for examples.

Each 3D PET image was a three-dimensional image consisting of 32–66 two-dimensional transaxial slices of 512 × 512 pixels on top of each other. In the disease recurrence classification challenge of this article, every transaxial PET slice is considered as an individual image. Binary masks were composed under specialist supervision to mark all the transaxial slices containing cancerous tissue. Both the PET images and the binary masks are presented in the NIfTI file format.

With the exception of one slice from one HNSCC patient, all slices showing the tumor of the positive patients were included in the study, which resulted in total 995 positive slices from the 89 HNSCC patients and 119 positive slices from the 11 patients with some other type of cancer. The corresponding numbers of negative slices were included in the data sets by choosing randomly 11–12 slices from 89 negative patients and 9–10 slices from the 11 other negative patients. We created our primary data set from the 995 positive HNSCC slices with equally many negative slices, and the additional test set consisted of the 119 positive non-HNSCC slices and the same number of negative slices. The primary data set was used to train and test models with five-fold cross-validation by dividing the data patient-wise into five possible test sets of 199 negative and 199 positive slices (exactly 20% of the total data set). Consequently, the non-augmented models were trained with a data set of 1592 slices and then tested with both a primary test set of 398 slices the additional test set of 238 slices.

### Software requirements

The preprocessing of the PET images and building of CNNs was done in Python (version: 3.9.4)^10^. In particular, the additional packages tensorflow (version: 2.6.0)^11^ and keras (version: 2.6.0)^12^ were used. The NIfTI images were loaded into Python with the package NiBabel (version: 3.2.1). The binary masks were created with Carimas^13^.

### Preprocessing

The original dimensions of the images were decreased from 512 × 512 to 128 × 128 because this considerably decreased the computational power and time needed for the training iterations and 128 × 128 was good enough resolution to detect the tumors in the images. The image size 128 × 128 has also been used to, for instance, detect lung cancer lesions from PET images^5^. The voxels of the original NIfTI images contained values ranging from 0 to approximately 19. To make the input more appropriate to be fed to a neural network, we scaled the images individually to the interval [0, 255] and rounded them into integers.

### Augmentation

In a half of the experiments performed, an augmentation routine was applied to the original training data to increase the number of images available for training the models. In both cases, the augmentation was used to multiply the size of the dataset by four. This was done by creating three new versions of each image by using clockwise and anticlockwise rotations of 15 degrees and a reflection against the sagittal plane so that the left and the right sides of a transaxial image were switched. The augmented training data set consisted of 6368 images.

### Convolutional neural network models

The methods used in this paper are classified as CNNs. More precisely, the models use matrix convolutions and pooling operations to automatically extract features from the input images. These features are then fed to a classical feedforward neural network which ultimately carries out the classification.

### Model configurations

**Figure 2:**
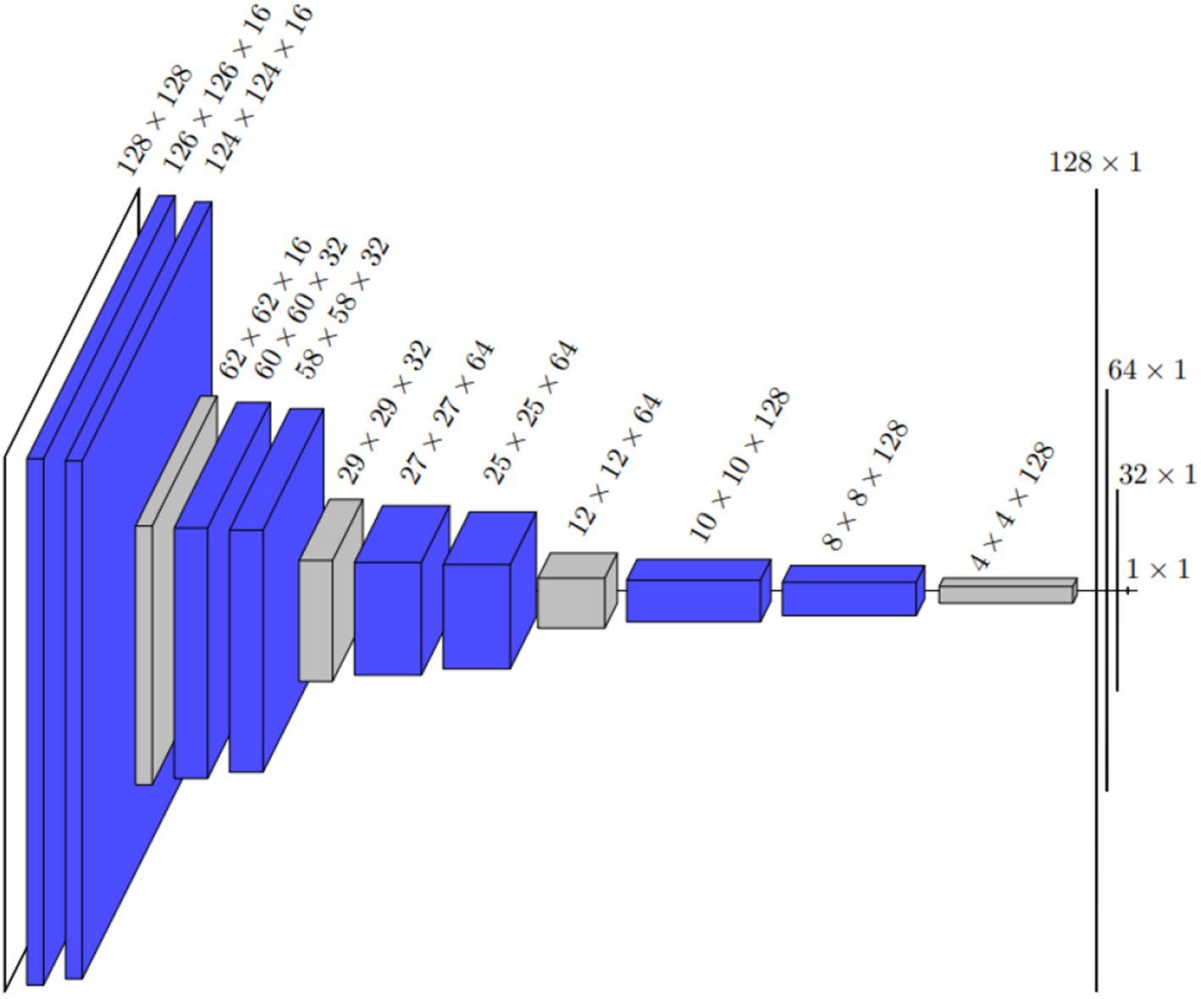
The architecture of the deep model drawn by showing the dimensions of each layer. After the first 128 × 128 input layer, all the blue layers are convolution layers, and all the gray layers are maximum pooling layers. The last three one-dimensional layers before the 1 × 1 output are dense layers. The total number of trainable parameters is 565,873.

**Figure 3:**
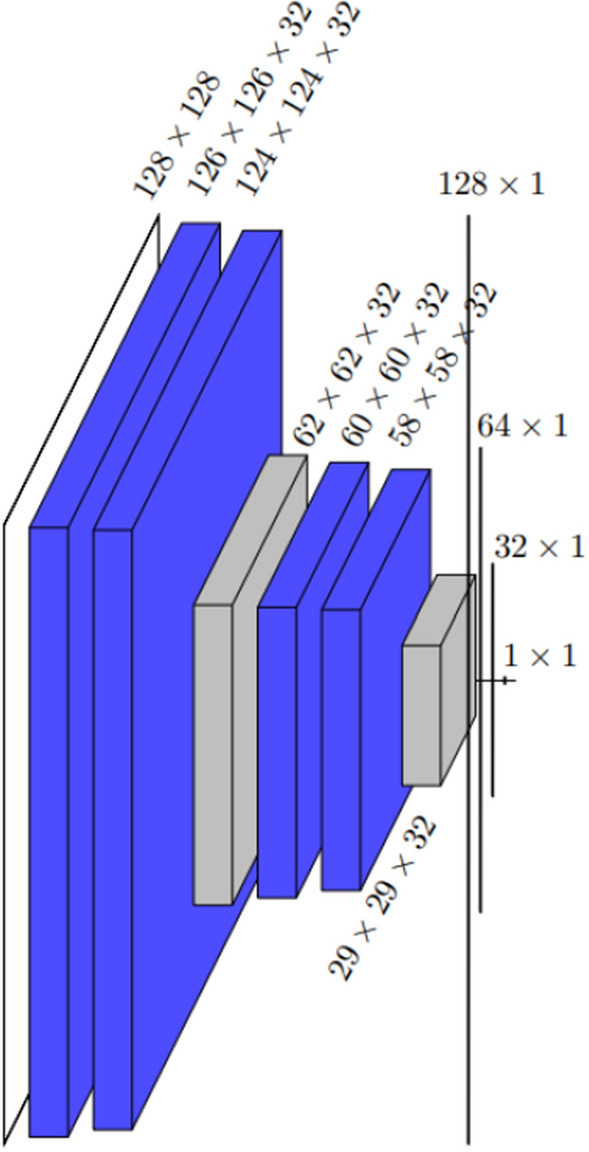
The architecture of the shallow model drawn by showing the dimensions of each layer. After the first 128 × 128 input layer, all the blue layers are convolution layers, and the two gray layers are maximum pooling layers. The last three one-dimensional layers before the 1 × 1 output are dense layers. The total number of trainable parameters is 3,483,297. The difference in the numbers of parameters of this CNN and the deep CNN is caused by the number of the first dense layer strongly depending on the dimension of the previous pooling layer.

The deep model architecture presented in this paper draws inspiration from the well-known U-Net architecture^14^. A typical U-Net consist of a contracting and an expanding path so that it first sees the whole image and is then able to focus on the details to perform segmentation. However, our CNNs have only the contracting path as it is designed for classification, not segmentation. The convolutions of the CNNs are performed in pairs, meaning that two convolutions are done back-to-back instead of one on each convolutional layer. After each pair of convolutions, a maximum pooling operation with a stride of 2 is used for down sampling. As seen from Fig. 2, 4 of these convolution-pooling sequences are done one after another in the deep model architecture. The size of convolutional kernel in all the convolution layers is 3 and no padding is used. The architecture is topped with a 3-layer feed forward and fully connected neural network with sigmoid-activation function on the output level. The second model considered in this paper, the shallow model with architecture presented in Fig. 3, only contains two pairs of convolutions followed by a pooling operation but the input ultimately flows to the dense part of the network similarly as in the deep model.

### Activation functions, loss function, and optimization parameters

Out of the two distinct activation functions used in the model architecture, the first one is the ReLU-function (Rectified Linear Unit):

$$ReLU\left(x\right)=\underset{ }{\mathrm{max}}\left\{0, x\right\}.$$

ReLU activations are widely applied in deep learning to introduce nonlinearity to the network. Clearly, the function is not differentiable at $x=0,$ but it is usually assumed that $ReL{U}^{^{\prime}}\left(0\right)=0$. ReLU-activations are mainly used in the hidden layers, and this is the case for the models inspected in this paper as well^15^.

Another rather popular activation function is the sigmoid function, also sometimes called the logistic function:

$$sigmoid\left(x\right)=\frac{1}{1+{e}^{-x}}.$$

The sigmoid function is normally used on the output level of a network in binary classification cases, as the values generated by the function can be interpreted as posterior probabilities^16^.

As is typical for binary classification models, binary cross-entropy was used as a loss function during the training process^17^. For two binary valued vectors $\widehat{{\varvec{y}}}$ and ${\varvec{y}}$ of length $N$, it is given by the formula

$$H\left({\varvec{y}}, \widehat{{\varvec{y}}}\right)=-\frac{1}{N}\sum_{i=1}^{N}{y}_{i}\mathrm{log}\widehat{{y}_{i}}+(1-{y}_{i})\mathrm{log}\left(1-\widehat{{y}_{i}}\right).$$

Based on preliminary tests, the number of epochs was initially fixed to 25 for all the models but the loss function was used for possible early stopping. This was done by comparing consecutive losses in the validation set that contained 30% of the training data. If there was no improvement in validation loss in 15 consecutive epochs, the model was assumed to have stopped learning and that number of epochs was ultimately used to train the model^18^. To minimize binary cross-entropy during the training process, stochastic gradient descent was used as a method of optimization. A constant learning rate of 0.001 was applied throughout every training iteration.

### Model evaluation

Confusion matrices were used to compute various statistics describing the performance of the models on the test set. By using the number of true positives (TP), true negatives (TN), false positives (FP) and false negatives (FN), we computed the following statistics to estimate the out-of-sample performance of the trained models:

- Binary accuracy: $$\frac{TP+TN}{TP+FP+TN+FN}.$$
- Sensitivity, also known as true positive rate (TPR): $\frac{TP}{TP+FN}.$
- Specificity: $$\frac{TN}{TN+FP}.$$

Additionally, we computed F1 scores using the formula

$$F1=\frac{2\bullet TPR\bullet PPV}{TPR+PPV},$$

where $PPV=\frac{TP}{TP+FN}$ is generally referred to as precision or positive predictive value^19^.

Furthermore, the ROC curves (Receiver Operating Characteristic) were constructed for determining the AUC values (Area Under receiver operator characteristic Curve) of each model.

### Binary classification

The output produced by the models for a single two-dimensional PET image was a real-valued estimate of the probability of cancer being present in PET image. As the goal of this research was to label these PET images as positive (contains cancer) or negative (does not contain cancer), it was necessary to find a suitable threshold for the classification. As the costs of false positive and false negative predictions were very hard to determine in this application, the intuitive classification threshold of 0.5 might not be suitable. Instead, Youden’s J-statistic was considered here.

Youden’s J-statistic with respect to some decision threshold τ can be formulated as follows:

$$J=\frac{TP\bullet TN-FN\bullet FP}{(TP+FN)(FP+TN)}.$$

As can be easily seen, this formula is equivalent to the sum of sensitivity and specificity minus 1. This can also be interpreted as the distance between the ROC curve of a randomly predicting model and the curve of the model of interest with respect to a given decision threshold. In particular, the ROC curve is often utilized to determine the value of the J-statistic in practice instead of the formula above. Regardless of the exact method, it is intuitive that by maximizing the value of the J-statistic, we achieve the best classification result^20^.

### Training and testing of the models

The deep and the shallow CNN introduced in Figs. 2 and 3 were applied to solve the classification problem. The data was given both CNN models with and without augmentation, resulting 4 different options: a non-augmented shallow model, an augmented shallow model, a non-augmented deep model, and an augmented deep model. Each model was trained with the primary data sets a total of 30 times so that there were five different iterations for each possible test set of five-fold cross-validation. These 30 iteration rounds were independent of each model because the model was re-initialized between them. After each training iteration, the Youden’s threshold was computed based on the predictions of the training data and the performance of the model was evaluated from the predictions of the primary test set given obtained from the cross-validation and the additional test set of different cancer types by using the metrics presented before.

### Statistical tests

To study the values of the evaluation metrics computed during the 30 iterations of the four model options, we use consider the p-values of the Wilcoxon signed-rank test, which is a popular non-parametric alternative of the Student’s t-test because, unlike the t-test, it suits for non-normally distributed data, such as values of our evaluation metrics.

### Approval for human experiments

All patients were at least 18 years old and gave informed consent to the research use of their data. All procedures performed in studies involving human participants were in accordance with the ethical standards of the 1964 Helsinki declaration. The study was approved by Ethics Committee of the Hospital District of Southwest Finland.

## Results

The median values of the evaluation metrics computed from the predictions of the test sets for the four models during the 30 iteration rounds are collected in Table 1. According to this table, the best model is the augmented deep model, followed by the non-augmented deep model and the augmented shallow model, while the non-augmented shallow model performs the poorest. The sensitivity of all the models is relatively low and this also affects accuracy and the F1 score of the models. In comparison, the specificity and AUC values are very good.

**Table 1:** Median values for the evaluation metrics computed from the predictions of the primary test sets during the 30 iteration rounds.

Model	Accuracy (%)	Sensitivity (%)	Specificity (%)	F1 (%)	AUC (%)
Non-augmented shallow	69.2	65.1	73.6	68.2	74.8
Augmented shallow	74.6	68.6	80.9	72.9	80.7
Non-augmented deep	74.7	70.4	77.6	73.6	82.2
Augmented deep	78.6	72.4	84.9	77.4	85.1

Table 2 summarizes the results of the Wilcoxon tests computed for six different model pairs from the values of the evaluation metrics in the primary test sets. While Table 1 shows that all the evaluation metrics except specificity have higher medians for the non-augmented deep model than the augmented shallow model, Table 2 reveals that the differences in these four evaluation metrics are not statistically significant. However, there is a statistically significant difference in the specificity of the non-augmented deep model and the augmented shallow model and this evaluation metric has a higher median in the case of the augmented shallow model. The differences in accuracy, specificity, F1 score, and AUC values are significant in accuracy between all the five other models pairs, so we can conclude for instance that the augmented deep model performs significantly better in terms of accuracy and AUC than the other models.

**Table 2:** p-values for the Wilcoxon signed-rank test between the evaluation metrics computed from the predictions of the primary test sets during the 30 iteration rounds. Statistically significant values with a 5% level of significance are written in bold.

Test	Accuracy	Sensitivity	Specificity	F1	AUC
Non-augmented vs augmented shallow	** < 0.001**	0.497	** < 0.001**	** < 0.001**	** < 0.001**
Non-augmented vs augmented deep	** < 0.001**	0.293	** < 0.001**	** < 0.001**	**0.00159**
Non-augmented shallow vs deep	** < 0.001**	0.251	** < 0.001**	**0.00225**	** < 0.001**
Augmented shallow vs deep	** < 0.001**	**0.00842**	**0.0104**	** < 0.001**	** < 0.001**
Augmented shallow vs non-augmented deep	0.0918	0.141	** < 0.001**	0.718	0.147
Non-augmented shallow vs augmented deep	** < 0.001**	**0.0367**	** < 0.001**	** < 0.001**	** < 0.001**

From Table 3, we can see what causes the low sensitivity of the models. Namely, this table contains the median values of sensitivity within such subgroups of the primary test sets that have been created based on the HNSCC tumor location of the patients. All the four models have higher median sensitivity for tumors on the root of the tongue, in fossa piriformis, in the oral cavity, and in the larynx than they have for the predictions of the whole test sets. However, their median sensitivity is lower when the tumor is either in the nasopharynx or in the oropharynx excluding the root of the tongue. The sensitivity is also high for unknown primary tumors and low in the case of other tumor locations. The other tumor locations include hypopharynx (excluding fossa piriformis), top of the esophagus, and a few other categories with very few patients.

**Table 3:** Different subgroups of positive head and neck squamous cell carcinoma patients based on the location of their tumor, the number of patients in the group, the total number of positive slices within the group, and median sensitivity (%) computed from the predictions of the primary test sets during the 30 iteration rounds. Values higher than the overall sensitivity in the primary test sets for the given model (see Table 1) are written in bold.

HNSCC tumor location	Patients	Slices in total	Non-augmented shallow	Augmented shallow	Non-augmented deep	Augmented deep
Oral cavity	18	203	**81.7**	**70.4**	**78.3**	**75.0**
Root of tongue	11	132	**96.8**	**86.7**	**97.7**	**83.3**
Oropharynx (exc. root of tongue)	13	141	50.8	33.3	51.1	35.4
Nasopharynx	8	83	33.3	26.1	39.4	45.2
Larynx	7	54	**65.8**	**73.7**	**73.7**	**77.0**
Fossa piriformis	5	90	**86.7**	**80.5**	**93.3**	**80.2**
Unknown primary	13	147	**90.6**	**88.5**	**90.6**	**88.5**
Other	14	145	50.0	55.8	55.2	67.7

Table 4 has the values of evaluation metrics computed from the additional test set containing images from positive patients that have some other head and neck cancer diagnosis than HNSCC. Compared to the Table 1, we see that our models perform slightly worse for this additional test than in the primary test sets of only HNSCC cases. In Table 5, we see that all the models detect follicular and papillary carcinoma of the thyroid gland and mucoepidermoid carcinoma of the parotid gland nearly perfectly but are unable to recognize chondrosarcoma of the neck. We also see that the augmented deep model has considerably lower sensitivity in the case of adenocystic carcinoma of the oral cavity, adenocarcinoma of the esophagus, and adnexal carcinoma of the skin than the other three models.

**Table 4:** Median values for the evaluation metrics computed from the predictions of the additional test set during the 30 iteration rounds.

CNN	Accuracy (%)	Sensitivity (%)	Specificity (%)	F1 (%)	AUC (%)
Non-augmented shallow	63.0	72.3	52.5	65.9	70.1
Augmented shallow	67.6	61.3	73.9	65.6	71.9
Non-augmented deep	70.4	73.5	67.6	70.7	78.1
Augmented deep	75.0	69.7	80.3	73.4	79.4

**Table 5:** Different subgroups of positive head and neck cancer patients based on their diagnosis, the number of patients in the group, the total number of positive slices within the group, and median sensitivity (%) computed from the predictions of the additional test set during the 30 iteration rounds. Values higher than the overall sensitivity in the additional test set for the given model (see Table 4) are written in bold.

Diagnosis	Patients	Slices in total	Non-augmented shallow	Augmented shallow	Non-augmented deep	Augmented deep
Adenocystic carcinoma of oral cavity	3	24	70.8	**70.8**	70.8	50.0
Adenocarcinoma of esophagus	2	29	**86.2**	**82.8**	**82.8**	69.0
Chondrosarcoma of neck	1	9	0.00	0.00	0.00	0.00
Papillary carcinoma of thyroid gland	2	33	**100**	**100**	**100**	**100**
Follicular carcinoma of thyroid gland	1	12	**100**	**100**	**100**	**91.7**
Mucoepidermoid carcinoma of parotid gland	1	5	**100**	**100**	**100**	**100**
Adnexal carcinoma of skin on neck	1	7	**100**	**100**	**100**	64.3

## Discussion

In this paper, we designed CNNs for classifying 2D slices of PET images. The reason behind focusing in 2D images instead of 3D images is that, in this way, all the slices in a 3D PET image could first be classified separately with the final CNN application and then these classifications can be considered together to see if they are reasonable or not. The classifications of 2D slices also gives information about the location and the length of the tumor, and training 2D models requires less data. Furthermore, while CNNs based on MRI and CT images have been very successful in the earlier research (see for instance^7^), we wanted to use only PET images here because the final models would then work regardless if the patient has a PET/CT or a PET/MRI scan.

All the models have a rather high level of specificity, which means that the models are generally able to classify negative slices correctly. On the other hand, their sensitivity levels are notably lower. The fact that the AUC values are higher than the values of the other metrics suggests that the Youden’s threshold is not ideal for converting continuous predictions into binary. Some sensitivity-weighted threshold could be more suitable. This issue seems to be present also in the similar research using deep learning to classify PET images. For example, Kirienko et al.^5^ developed CNNs for the classification of the ^18^F-FDG PET images of lung cancer patients and obtained accuracy of 90% but sensitivity of only 47%. We might have obtained higher overall accuracy here if we would have included all the negative slices in our data but, since this would likely been on the cost of sensitivity, we trained and predicted balanced data sets with equally many negative and positive instances.

According to the predictions from the primary test sets, the augmentation improves the results of both shallow and deep CNNs. This conclusion about data augmentation is very expected and in line with results from other publications with similar experimental objectives. For example, Xu et al.^8^ made similar observations on the use of augmented data when it comes to binary cancer classification from two-dimensional gray scale images as augmentation increased the values of the evaluation metrics by a few percent points also in their research. Furthermore, the deep models work generally better than the shallow models, which was also to be expected.

The sensitivity of the models also differs much based on the location of the primary head and neck tumor. The models have highest sensitivity for tumors on the root of tongue and in the fossa piriformis, which is likely because these locations are very specific and there are enough patients in these subgroups. The fact that the models have low sensitivity for the subgroup of other locations is likely because this group contains such tumor locations that only one or two patients have and, since the data is divided patient-wise into test and training sets, the models do not have enough data to learn these cancer types on the iteration rounds where they are in the test set. The poor sensitivity for tumors in the nasopharynx might be because high ^18^F-FDG concentration in the brain hinders recognizing the nearby tumors.

We also noticed that the models work slightly worse in the additional test set than in the primary test set. It was expected that models trained on HNSCC data work better for this cancer type but the fact that the difference in the medians of the evaluation metrics between the test sets is only a few percent points is promising. Namely, this suggest that we could use transfer learning since the models trained on HNSCC data require less new data to learn to detect some other head and neck cancer types. Especially, our results give evidence that this would work if we wanted models that detect cancer in the thyroid or the parotid glands.

One interesting notion is that the augmented deep model has lower sensitivity than the other models for especially in certain subgroups of the additional test set. While it has the highest median accuracy and AUC for the predictions in both the primary and the additional test sets, we see that it is better mostly because its specificity is higher than that of the three other models. Especially, it detects worse adenocystic carcinoma of the oral cavity, adenocarcinoma of the esophagus, and adnexal carcinoma of the skin on neck. The interpretation of this result can be seen in different ways, depending whether it is preferable that a model trained on solely HNSCC images classifies only them as positive or that it also detects other cancer types.

The image data set used in this paper introduces some limitations to the final models. The transaxial PET slices are very heterogenous as slices from the different parts of the head and neck area notably differ from each other even within the PET image of one patient. Considering this fact, the data set used here is quite small. While the size of the data set could be increased using even more augmentation, it should be carefully considered which modifications are realistic. For instance, we used here such reflections that switch the right and the left side of the transaxial images because the bilateral symmetry of the human body. The reflections in the other direction were not used since the shape of jaw and brains considerably differs between the front and the back of the head. For similar reasons, rotations of higher number of degrees and translations were not performed, and the images were not blurred and no noise was added because the PET images are not sharp in the same way as MRI and CT images. However, new augmentation techniques such as generative adversarial networks could potentially work here if there is no data from greater number of patients available.

## Conclusion

It is evident that combining the PET imaging together with machine learning techniques, such as CNNs, can help the doctors to find cancer tumors. In this study, different CNNs were used to classify the patients based on their images containing head and neck cancer or not. According to our results, a deep CNN model with data augmentation is the most suited design for this task in terms of the AUC value. It has high specificity, and its sensitivity is especially good for tumors in the oral cavity, on the root of the tongue, and in the fossa piriformis.

## Data Availability

The datasets used and/or analysed during the current study available from the corresponding author on reasonable request.

## Code availability

The code used to train and evaluate the models presented in this paper is available as a public repository on Github: https://github.com/hmhellstrom/Cancer_classification.git.

## References

[1] National Institutes of Health (NIH). *Head and Neck Cancers *(2021).

[2] Vigneswaran N, Williams MD (2014). Epidemiologic trends in head and neck cancer and aids in diagnosis. Oral Maxillofac. Surg. Clin. N. Am..

[3] Global Cancer Observatory (GCO). *Cancer Today [Online Analysis Table]* (2022).

[4] Ziegler SI (2005). Positron emission tomography: Principles, technology, and recent developments. Nucl. Phys. A.

[5] Kirienko M, Sollini M, Silvestri G, Mognetti S, Voulaz E, Antunovic L (2018). Convolutional neural networks promising in lung cancer T-parameter assessment on baseline FDG-PET/CT. Contrast Media Mol. Imaging.

[6] Pinochet P, Eude F, Becker S, Shah V, Sibille L, Toledano MN, Modzelewski R, Vera P, Decazes P (2021). Evaluation of an automatic classification algorithm using convolutional neural networks in oncological positron emission tomography. Front. Med..

[7] Wang W, Charkborty G (2021). Automatic prognosis of lung cancer using heterogeneous deep learning models for nodule detection and eliciting its morphological features. Appl. Intell..

[8] Xu, Q., Wang X., Jiang, H. Convolutional neural network for breast cancer diagnosis using diffuse optical tomography. (2019). 10.1186/s42492-019-0012-y. 10.1186/s42492-019-0012-y PMC7099566 32240400

[9] Yang C-K, Yeh JC-Y, Yu W-H, Chien L-I, Lin K-H, Huang W-S, Hsu P-K (2019). Deep convolutional neural network-based positron emission tomography analysis predicts esophageal cancer outcome. J. Clin. Med..

[10] van Rossum, G., & Drake, F. L. *Python 3 Reference Manual*. CreateSpace (2009).

[11] Abadi, M., Agarwal, A., Barham, P., Brevdo, E., Chen, Z., Citro, C. *et al*. *TensorFlow: Large-Scale Machine Learning on Heterogeneous Systems* (2015).

[12] Chollet, F. *et al*. *Keras*. (GitHub, 2015).

[13] Rainio, O., Chunlei, H., Teuho, J., Nesterov, S.V., Oikonen, V., Piirola, S. *et al*. Carimas: An extensive medical imaging data processing tool for research. *J. Digit. Imaging*. 10.1007/s10278-023-00812-1 (2023). 10.1007/s10278-023-00812-1 PMC10406992 37106213

[14] Ronneberger, O., Fischer, P., & Brox, T. *U-Net: Convolutional Networks for Biomedical Image Segmentation* 234–241 (2015). 10.1007/978-3-319-24574-4_28

[15] Goodfellow I, Bengio Y, Courville A (2016). Deep Learning.

[16] Alpaydin, E. *Introduction to Machine Learning*, 3rd ed. (MIT Press, 2014).

[17] Murphy, K. P. *Machine Learning: A Probabilistic Perspective* (MIT Press, 2012).

[18] *tf.keras.callbacks.EarlyStopping*. TensorFlow (2021).

[19] Pedregosa F, Varoquaux G, Gramfort A, Michel V, Thirion B, Grisel O (2011). Scikit-learn: Machine learning in Python. J. Mach. Learn. Res..

[20] Youden WJ (1950). Index for rating diagnostic tests. Cancer.

